# Allogenic and autologous anti-CD7 CAR-T cell therapies in relapsed or refractory T-cell malignancies

**DOI:** 10.1038/s41408-023-00822-w

**Published:** 2023-04-25

**Authors:** Yinqiang Zhang, Chenggong Li, Mengyi Du, Huiwen Jiang, Wenjing Luo, Lu Tang, Yun Kang, Jia Xu, Zhuolin Wu, Xindi Wang, Zhongpei Huang, Yanlei Zhang, Di Wu, Alex H. Chang, Yu Hu, Heng Mei

**Affiliations:** 1 Institute of Hematology, Union Hospital, Tongji Medical College, Huazhong University of Science and Technology, Wuhan, 430022 China; 2 Hubei Clinical Medical Center of Cell Therapy for Neoplastic Disease, Wuhan, 430022 China; 3 Shanghai YaKe Biotechnology Ltd, Shanghai, China; 4 Beijing GoBroad Hospital Management Co. Ltd, Beijing, China; 5 Clinical Translational Research Center, Shanghai Pulmonary Hospital, Tongji University School of Medicine, Shanghai, China

**Keywords:** Cancer immunotherapy, Acute lymphocytic leukaemia, T-cell lymphoma

## Abstract

Chimeric antigen receptor-T (CAR-T) therapy remains to be investigated in T-cell malignancies. CD7 is an ideal target for T-cell malignancies but is also expressed on normal T cells, which may cause CAR-T cell fratricide. Donor-derived anti-CD7 CAR-T cells using endoplasmic reticulum retention have shown efficacy in patients with T-cell acute lymphoblastic leukemia (ALL). Here we launched a phase I trial to explore differences between autologous and allogeneic anti-CD7 CAR-T therapies in T-cell ALL and lymphoma. Ten patients were treated and 5 received autologous CAR-T therapies. No dose-limiting toxicity or neurotoxicity was observed. Grade 1–2 cytokine release syndrome occurred in 7 patients, and grade 3 in 1 patient. Grade 1–2 graft-versus-host diseases were observed in 2 patients. Seven patients had bone marrow infiltration, and 100% of them achieved complete remission with negative minimal residual disease within one month. Two-fifths of patients achieved extramedullary or extranodular remission. The median follow-up was 6 (range, 2.7–14) months and bridging transplantation was not administrated. Patients treated with allogeneic CAR-T cells had higher remission rate, less recurrence and more durable CAR-T survival than those receiving autologous products. Allogeneic CAR-T cells appeared to be a better option for patients with T-cell malignancies.

## Introduction

Chimeric antigen receptor-T (CAR-T) cell therapy provides an emerging and promising option for patient with hematological malignancies. CAR-T cells targeting CD19 have shown excellent clinical results with a complete remission (CR) rate of 90% in B-cell acute lymphoblastic leukemia (B-ALL) [1–4]. To date, 5 CAR-T products have been approved by Food and Drug Administration and 2 have been marketed in China. T-cell malignancies are heterogeneous diseases. T-ALL accounts for 15–25% in ALL [5]. Relapse remains a challenge in T-ALL and indicates a poor prognosis [6]. In non-Hodgkin lymphoma, approximately 15% of patients are diagnosed with T-cell lymphoma [7]. Disease refractoriness is common in T-cell lymphoma and correlates with dismal outcomes [8–10]. Novel therapies are needed for these patients.

CAR-T cell therapies for T-cell malignancies have been explored and their targets include CD7, CD5, CD99 and CD38 [11–13]. CD7 is a transmembrane glycoprotein highly expressed in T-cell lymphoblastic leukemias and lymphomas (>95%), and is also present on the majority of T cells, NK cells, and their precursors [14–16]. Thus, CAR-T cells targeting CD7 have the potential to eliminate tumor cells, but also holds the risk to kill normal T and CAR-T cells. Since CD7 is proven non-essential for T-cell development or function [17, 18], several strategies including CRISPR-Cas9, natural selection, and endoplasmic reticulum retention, have been developed to block CD7 expression on CAR-T cells [19–22]. By adding a vector containing a CD7-binding domain fused to an endoplasmic reticulum retention signal domain, CD7 trafficking to the cell surface was restrained, thus avoiding fratricide among anti-CD7 CAR-T cells [22]. The IntraBlock donor-derived anti-CD7 CAR-T cells have demonstrated great safety and efficacy in patients with T-ALL in a phase I clinical trial [22]. However, it offers us space to explore the use of allogeneic or autologous cells.

Peripheral blood mononuclear cells (PBMCs) from patients are easy to acquire and are not limited to appropriate donors. Autologous CAR-T cells do not cause severe graft-versus-host disease (GVHD). For patients who have not exposed to or do not plan to bridge allogeneic stem cell transplantation (SCT), autologous CAR-T cells can avoid rejection by autoimmune system. Yet, it remains a challenge to distinguish between normal T and malignant T cells. Circulating malignant T cells have the risk to contaminate CAR-T cell products [23]. Moreover, T cells from patients with heavily pretreated diseases have inferior quality and quantity. To investigate the effects of different cell sources, we launched a phase I clinical trial to evaluate the safety and efficacy of anti-CD7 CAR-T cells in adolescents and adults with refractory or relapsed (r/r) T-cell malignancies, and further compared the differences between autologous and allogeneic CAR-T cells.

## Methods

### Clinical trial design

This is a single-center phase I study of anti-CD7 CAR-T cell therapy in patients with r/r T-cell malignancies (NCT04823091). Eligible Patients must age 14–70 years old, be diagnosed with CD7-positive r/r T-cell ALL or lymphomas [24], with Eastern Co-operative Oncology Group status of 0–2, and without un-controllable infections, active intracranial lesions or organ failures. All patients have provided written informed consents. The trial was approved by the Medical Ethics Committee of the Union Hospital affiliated to Huazhong University of Science and Technology, Wuhan, China.

### CAR-T cell production and patient treatment plan

Peripheral blood mononuclear cells (PBMCs) were collected from patients or donors. Donors must be equal to or over 5/10 HLA-identical siblings or 10/10 HLA-matched unrelated donors. The production process was detailed as previously described [22]. Patients received cyclophosphamide 300 mg/m^2^ and fludarabine 30 mg/m^2^ daily on day −5 to −3 and infusion of anti-CD7 CAR-T cells at the dose of 1 or 2 × 10^6^ CAR-T cells/kg on day 0.

### End points

Primary end points included incidence of treatment-related adverse events. Cytokine release syndrome (CRS) and immune effector cell-associated neurotoxicity syndrome were graded according to ASTCT consensus [25]. GVHD was graded according to International Bone Marrow Transplant Registry severity index [26]. Organotoxicities, hematologic toxicities, and infections, were graded according to CTCAE, version 5.0 (http://ctep.cancer.gov/). If the grade of hematological toxicity raised after the infusion compared to that after lymphodepletion and before infusion, it is considered as a CAR-T associated adverse effect. Secondary end points included clinical responses and kinetics of CAR-T cells. Bone marrow (BM) blasts were assessed by flow cytometry (FCM) and cell morphology on day 7, 14 and 21 after infusion. Minimal residual disease (MRD) negativity was defined as less than 0.01% nucleated cells. Extramedullary diseases (EMD) were measured by imaging including positron emission tomography-computed tomography (PET-CT) and/or CT. Proliferation and duration of in vivo CAR-T cells were detected by FCM and droplet digital polymerase chain reaction (ddPCR). Serum cytokines were detected by FCM using cytometric bead array as previously described [27].

### Collection and loading of single-cell RNA-seq samples

PBMCs were isolated by Ficoll density gradient sedimentation and then captured using the SeekOne^®^ DD Single Cell 5′ library preparation kit following the manufacturer protocol. Libraries sequencing was performed in an Illumina NovaSeq 6000. The specific protocol is detailed in the supplementary materials.

### Statistical analysis

Clinical safety, efficacy and CAR-T kinetics were summarized and analyzed using descriptive statistics. All analyses were performed with the use of GraphPad Prism 9.0. Correlation and subgroup analysis were done by the two-tailed student’s *t* test. *P* < 0.05 was considered statistically significant.

Single-cell RNA-seq data were analyzed by R package Seurat. Reciprocal Principal Component Analysis was administrated to integrate two samples. Cells were clustered based on a graph-based clustering approach, and were visualized in 2-dimension using Uniform Manifold Approximation and Projection (UMAP). Significant differentially expressed genes were identified as log_2_ average expression >1 and *P* < 0.00001.

## Result

### Characteristics of patients and anti-CD7 CAR-T infused products

**Fig. 1:**
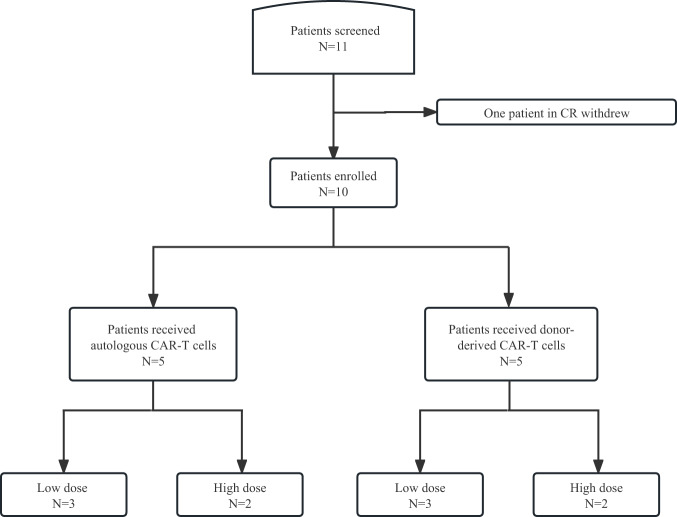
Consort diagram of patient flow. Ten patients were enrolled in the study and underwent leukapheresis. Anti-CD7 CAR-T cells were derived from patients or their donors and infused into all patients at a low dose (1 × 10^6^/kg, *n* = 5) or a high dose (2 × 10^6^/kg, *n* = 5).

From April 15, 2021 to January 13, 2022, 11 patients were enrolled in this trial. Expect for one patient who achieved complete remission (CR) before CAR-T cell infusion, 10 were treated (Fig. 1). Seven patients (70%) were male, and the median age was 32 (range, 16–69) years (Table 1). All enrolled patients had CD7^+^ T-cell malignancies including adult T-ALL, T-cell lymphoblastic lymphoma, angioimmunoblastic T-cell lymphoma and mycosis fungoides. The median lines of previous therapies were 4 (range, 2–10). One patient received an excision of mediastinal tumor, 7 patients received allogeneic SCT, and one received donor lymphocyte infusion. Five patients (50%) had ALL including one with extramedullary disease (EMD) involving the nasopharynx and clavicle. Four patients were diagnosed with lymphoma grading II-IV according to Ann Arbor stage. Two of them had BM infiltrations and four had extranodular lymphoma including mediastinum, lung, chest wall, gastrointestinal tract, spleen, and subcutaneous tissue. Patient 1 had IIIA mycosis fungoides staging by TNMB (tumor, node, metastasis, blood) classification [28]. The median blasts were 25% (range, 0.42–53%) in 7 patients with BM infiltration. Three patients had high-risk mutations including RUNX1 [29, 30], HOX11L2 [31], and KMT2D [32]. Patient 6 and patient 7 received chidamide, a histone deacetylase inhibitor, as bridging therapy after aphesis and before lymphodepletion.

**Table 1:** Baseline patient characteristics.

Characteristics	Total (*N* = 10)	Autologous (*N* = 5)	Donor derived (*N* = 5)
Median age (range), years	32 (16–69)	32 (16–69)	23 (18–36)
Male, No. (%)	7 (70)	3 (60)	4 (80)
Prognosis of disease, No (%)			
Acute T-cell lymphoblastic leukemia	5 (50)	2 (40)	3 (60)
T-cell lymphoblastic lymphoma	3 (30)	1 (20)	2 (40)
Angioimmunoblastic T-cell lymphoma	1 (10)	1 (20)	0 (0)
Mycosis fungoides	1 (20)	1 (20)	0 (0)
Previous therapies			
Median lines of therapy (range)	4 (2–10)	4 (2–4)	5 (4–10)
Surgery, No (%)	1 (10)	1 (20)	0 (0)
Allogeneic SCT, No (%)	6 (60)	2 (40)	4 (80)
Donor lymphocyte infusion, No (%)	1 (10)	0 (0)	1 (20)
Primary refractory disease, No (%)	4 (40)	3 (60)	1 (20)
Baseline disease burden			
Bone marrow blasts, %			
>50	1 (10)	1 (20)	0 (0)
25–50	3 (30)	1 (20)	2 (40)
5–25	2 (20)	0 (0)	2 (40)
<5	4 (40)	3 (60)	1 (20)
Median blasts, range	15 (0–53)	0 (0–25)	40 (15–53)
EMD, No (%)	5 (50)	3 (60)	2 (40)
High-risk phenotype or genotypes	4 (40)	2 (40)	2 (40)
≥Grade 3 cytopenia, No (%)	6 (60)	4 (80)	2 (40)
Bridging therapies	2 (20)	1 (20)	1 (20)

*SCT* stem cell transplatation, *EMD* extramedullary disease.

Based on the patient’s tumor burden, pancytopenia status, the preference and donor availability, PBMCs were collected from the patients (*n* = 5) or donors (*n* = 5) for CAR-T cell production. The characteristics of infused CAR-T cell products are presented in Supplementary Table 1. The median transfection efficiency was 46.3% (range, 40.2–81.7%) and the median CD4^+^/CD8^+^ T-cell ratio was 6.6 (range, 1.5–55.9). All cell products were frozen due to long-distance transportation and were thawed within 15 min before infusion. The median vein-to-vein time of patients with autologous CAR-T cells was 18 days (range, 16–26), and that of patients with allogeneic CAR-T cells was 22 days (range, 17–101). Due to various reasons such as patients’ infections, overloaded ward and COVID-19, the vein-to-vein time was generally longer than the manufacturing time (Supplementary Table 2). One patient achieved CR when the product was done and receiving allogeneic CAR-T cells after his second relapse.

### Safety

Eight patients (80%) experienced CRS, with grade 1–2 in 7 patients (70%) and grade 3 in one patient (10%) (Table 2). The median time of onset and duration of CRS was 10 (range, 7–15) days and 4 (range, 2–9) days, respectively. Both tocilizumab and corticosteroids were administrated for 5 patients. Considering the serum levels of interleukin-6, 3 patients received dexamethasone alone (Supplementary Table 3) [33]. Immune effector cell-associated neurotoxicity syndrome was not observed among these participants. Two patients were diagnosed with hemophagocytic lymphohistiocytosis (HLH). Patient 3 recovered from HLH after intravenous steroids. Steroids and etoposide failed to control Epstein-Barr virus (EBV)-related HLH of patient 10, and the patient died from fungal pneumonia on day 63.

**Table 2:** Adverse events within a month post infusion.

Adverse events	Grade 1	Grade 2	Grade 3	Grade 4	Total patients
CRS					
Total score	3 (30)	4 (40)	1 (10)	0	8 (80)
Fever	3 (30)	4 (40)	1 (10)	0	7 (70)
Hypoxia	0	3 (30)	1 (10)	0	4 (40)
Hypotension	0	2 (20)	1 (10)	0	3 (30)
ICANS					
Total score	0	0	0	0	0
GVHD					
Total score	1 (10)	1 (10)	0	0	2 (20)
Skin	1 (10)	1 (10)	0	0	2 (20)
Intestinal	0	1 (10)	0	0	1 (10)
Liver	0	0	0	0	
Hematological Event					
Anemia	1 (10)	0	3 (30)	0	4 (40)
Thrombocytopenia	2 (20)	1 (10)	1 (10)	5 (50)	4 (40)
Leukopenia	0	0	0	9 (90)	9 (90)
Neutropenia	0	0	0	9 (90)	9 (90)
Lymphocytopenia	0	0	0	9 (90)	9 (90)
Infection					
Total scores	0	0	5 (50)	0	4 (40)
Virus	0	0	1 (10)	0	1 (10)
Bacteria	0	0	3 (30)	0	3 (30)
Fungus	0	0	1 (10)	0	1 (10)
Others					
Hypofibrinogenemia	5 (50)	0	0	0	5 (50)
Capillary leak syndrome	2 (20)	0	0	0	2 (20)
HLH	0	0	1 (10)	1 (10)	2 (20)

*CRS* cytokine release syndrome, *ICANS* immune effector cell-associated neurotoxicity syndrome, *GVHD* graft-versus-host disease, *HLH* hemophagocytic lymphohistiocytosis.

GVHD of grade 1–2 occurred in two patients. Patient 4 who received allogeneic CAR-T cells developed acute GVHD presented as diarrhea and maculopapular rash. Patient 2 had previously received allogeneic SCT. She was treated with autologous CAR-T cells and developed chronic GVHD characterized as skin desquamation and pigmentation. GVHDs of these patients were well controlled by tacrolimus and methylprednisolone.

Pancytopenia was generally observed in these patients which might correlate to patients’ marrow reserves, lymphodepletion or CAR-T therapy (Supplementary Table 4). Five patients (50%) had grade ≥3 cytopenia before lymphodepletion. All patients developed grade 4 lymphopenia, neutropenia and leukopenia after infusion. Persistent grade 3–4 lymphopenia and neutropenia over one month occurred in 2 patient and 3 patients, respectively. Secondary appearance of grade 4 lymphopenia and neutropenia were observed in patient 2 and 10. Patient 4 developed grade 4 lymphopenia during day 52 to day 75. Seven patients (70%) suffered grade ≥ 3 thrombocytopenia. Grade ≥ 3 anemia occurred in 7 patients (70%). A significant secondary decrease of platelets was found in patients 4, 8 and 9 (Supplementary Fig. 1). Patient 9 recovered to an absolute platelet count of at least 50,000 per cubic millimeter by day 28 but plunged to 21,000 per cubic millimeter on month 2. The patient died from a sudden intracerebral hemorrhage on day 81 and the pathogenesis was unclarified.

Six patients experienced 9 infections (Supplementary Table 3). Five occurred within one month after infusion, with 3 bacterial infections, 1 fungal infection and 1 viral infection. Three patients suffered from cytomegalovirus (CMV) or EBV activation, manifested with cough, fever or diarrhea. Patient 4 developed viral pneumonia associated with EBV infection on day 62 and died from respiratory failure on day 75 (Supplementary Table 5).

### Efficacy and long-term outcomes

**Fig. 2:**
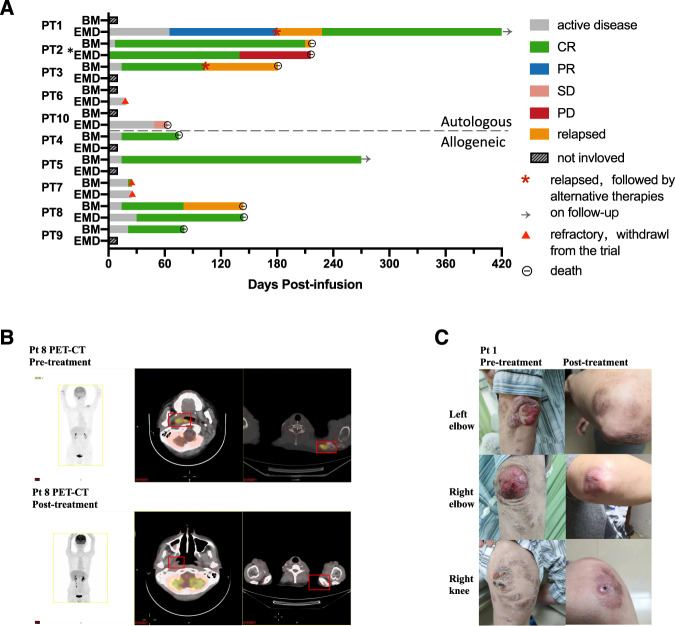
Clinical response to anti-CD7 CAR-T cells. **A** The clinical responses of patients’ BM and EMD post anti-CD7 CAR-T infusion are shown by swimmer plots. For patients without BM infiltration or EMD, striped bars are presented. PT patient, BM bone marrow, EMD extramedullary disease, CR complete remission, PR partial response, SD stable disease, PD progressive disease. * EMD did not existed during screening in patient 2 and she achieved MRD- CR after infusion. On the 140th post infusion, extramedullary recurrence occurred in the gastric wall, pelvic peritoneum and multiple lymph nodes detected by PET-CT. **B** Representative PET-CT images of patient 6 before and after anti-CD7 CAR-T cells infusion. As is shown in PET-CT, patient 6 had lesions in nasopharynx and left clavicle. The patient achieved extramedullary complete remission at day 30 post infusion. **C** Images of patient 1 before and after anti-CD7 CAR-T cells infusion. Patient 1 had diffuse lesions over the body, especially on his elbows and right knee (left), and achieved remarkable remission with a small phyma on the right knee on Day 65.

At a median follow-up of 6 (range, 2.7–14) months, the best overall response rate (ORR) was 70% (Fig. 2A). Among 7 responders, 3 patient received autologous cells and 4 were treated allogeneic therapies. Within a month post infusion, 7 patients with BM infiltration (100%) achieved MRD-negative CR. Four patients with high-risk mutations (100%) achieved molecular remission. Two patients with EMD or extranodular infiltrations responded to CAR-T therapies, including 1 CR (Fig. 2B) and 1 partial remission (PR) (Fig. 2C). Patients 6 and 7 with progressively bulky lymphoma withdrew from the trial on day 14 and 25, respectively. Shrunken lymphadenopathy was observed in patient 10 on day 49, but unfortunately, the reduction did not reach the criteria of PR.

None of patients received SCT after CAR-T cell therapies. After autologous CAR-T cell therapies, patient 1 with mycosis fungoides achieved PR on day 65, relapsed on day 180 and attained CR followed by 11-fraction radiotherapy; patients 2 and 3 suffered CD7^+^ relapses on day 103 and 140, respectively. After allogeneic CAR-T cell therapies, patient 8 with KMT2D and HOX11L2 mutations had a CD7^-^ relapse at month 3; patient 5 with T-ALL maintained MRD-negative remission for 9 months; patients 4 and 9 died during remission as previously described.

### Kinetics of anti-CD7 CAR-T cells and serum cytokines

**Fig. 3:**
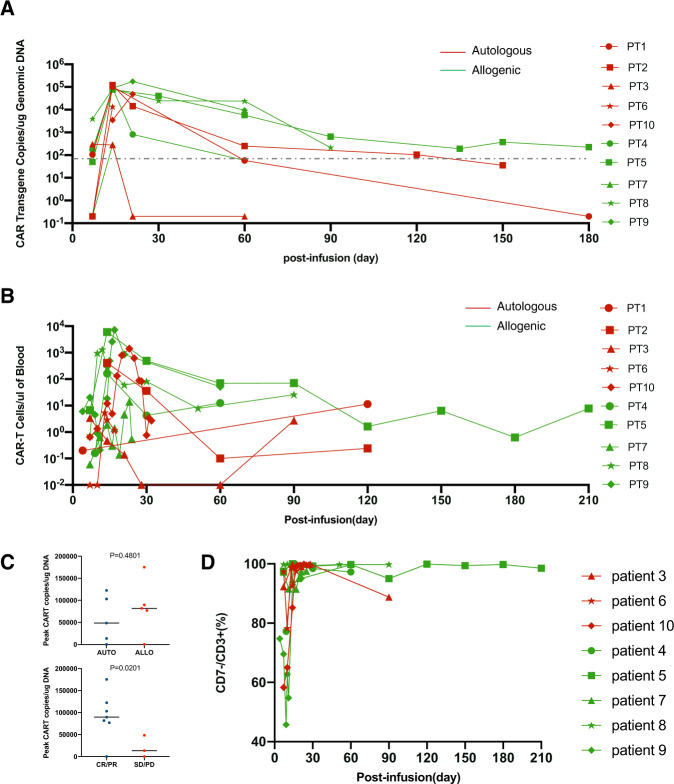
CAR-T cell expansion and persistence. **A** Kinetics of CAR vector transgene copies per microgram of genomic DNA of peripheral blood mononuclear cells in individual patients measured by ddPCR. The dotted line indicated the threshold determined by quantitation of normal samples. **B** Kinetics of CD7- CAR-T cells in peripheral blood detected by FCM. **C** Correlation of the peak CAR copies in the peripheral blood with sources and efficacy. **D** The percentages of CD7^+^ T cells were determined by FCM. Due to the lack of specimens, the condition of patients 1 and 2 could not be detected.

We adopted two methods to detect in vivo CAR-T cells. During treatment, FCM is more convenient to trace the expansion of CAR-T cells. However, PCR has higher sensitivity for tracking a trickle of CAR-T cells during follow-up. The median time of peak expansion measured by FCM was 14 (range, 7–23) days after infusion. The median levels of peak expansion were 409.0 (range,11.4–8640.0) per μl measured by FCM and 7.95 × 10^4^ (range, 2.88 × 10^2^–1.75 × 10^5^) copies per microgram genomic DNA measured by ddPCR, respectively (Fig. 3A, B). Limited by the small sample size, peak CAR copies were not significantly correlated with cell sources, disease subtypes, tumor burden and dose of infused cells, but they were associated with the best efficacy (*P* = 0.02) (Fig. 3C, Supplementary Fig. 2). Four patients (57.1%) had a relatively high level of CAR-T copies detected by ddPCR at month 2, among whom 3 received allogeneic CAR-T cells and 1 received autologous products.

**Fig. 4:**
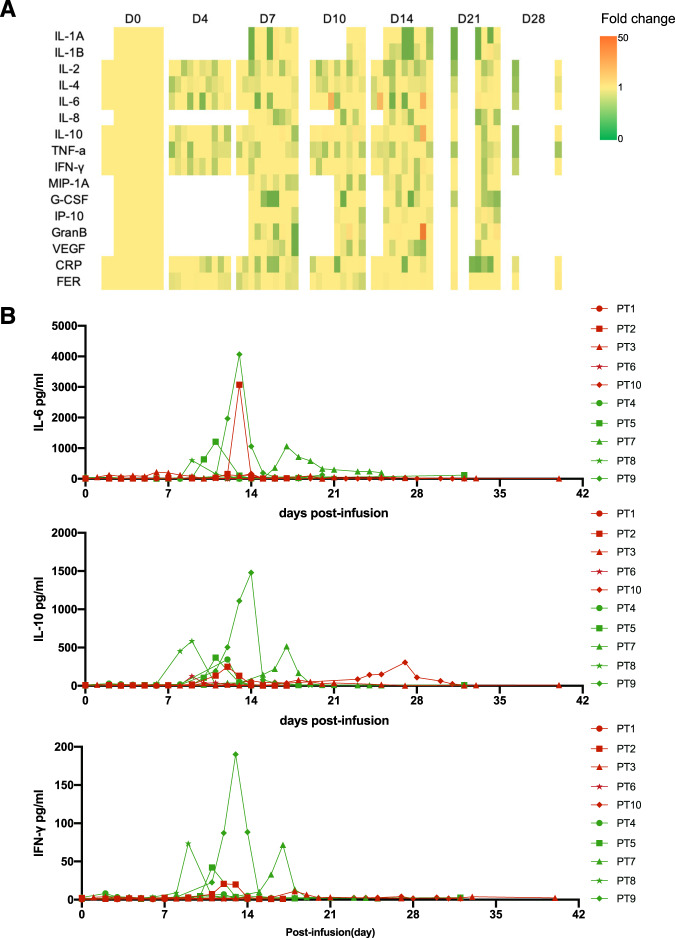
Serum biomarkers in peripheral blood after CAR-T cell infusions. **A** A heatmap of biomarkers. Each row is a biomarker, and each column is a time point. At each time point, the color patches from left to right represent patient 1–10, respectively. **B** Kinetics of IL-6, IL-10 and IFN -γ in peripheral in individual patients measured by cytometric bead array. Pronounced increases of these cytokines were detected in patients 2, 5, 7, 8 and 9, which were overlapped with grade 1 or 2 cytokine release syndrome and precede the peak of CAR-T cell expansion.

Sixteen serum biomarkers were detected after infusions, among which 10 rise at different levels in a group of patients (Fig. 4A). Unfortunately, the data was insufficient for statistical analysis. Serum interleukin-6, interleukin-10, interferon-γ showed significant summits in patients 2, 5, 7, 8 and 9 (Fig. 4B). These summits clung to the occurrence of CRS-related symptoms and were prior to CAR-T cell peak amplification (Supplementary Fig. 3).

The absolute count of T cells had a transient decline after CAR-T infusion followed by a dramatic rise due to CAR-T cell proliferation (Supplementary Fig. 4). Accompanied by CAR-T amplification, CD7^+^ T cells were rapidly eliminated and CD7^−^ cell subsets remained alive (Fig. 4D). T cells reached a median count of 401.82 (range, 46.47–1998.40) per μl a month post infusion.

### Single-cell RNA sequencing

**Fig. 5:**
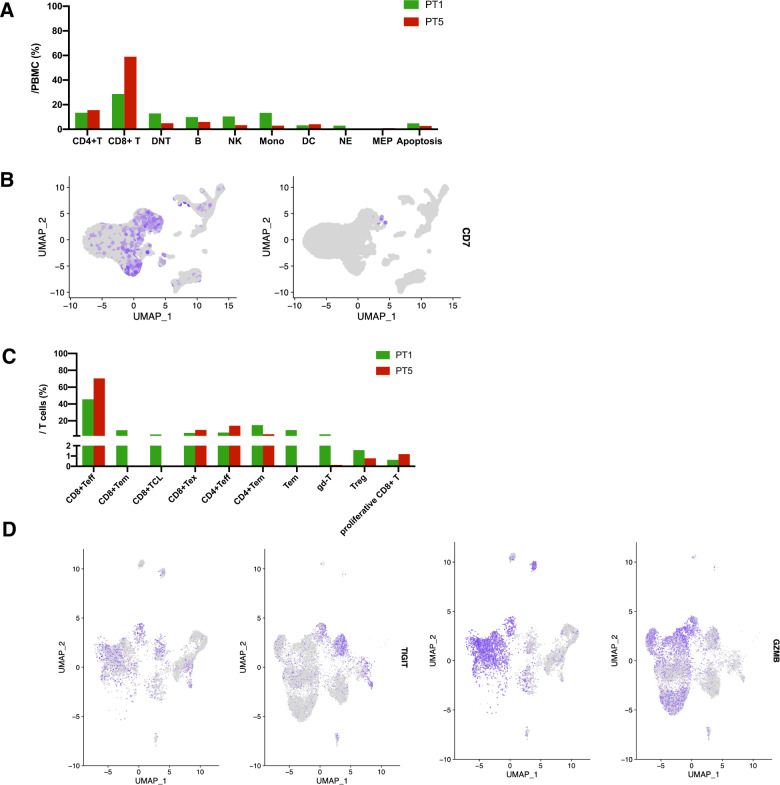
Single-cell RNA sequencing of patients 1 and 5. **A** Proportion of various types of cells in peripheral blood mononuclear cells (PBMCs) from patient 1 and patient 5. **B** Visualize the expression of CD7 in peripheral blood cells by UMAP plots. **C** Proportion of various types of cells in T cells (PBMCs) from patient 1 and patient 5. **D** Visualize the expression of TIGHT and GZMA in T cells by UMAP plots. Patient 1 (left), patient 5 (right).

Patients 1 and 5 achieved durable remission after receiving CAR-T cell therapies. To further explore the immune reconstitution after CAR-T cell infusion, we performed single-cell transcriptomic sequencing of PBMCs derived from the two patients. Sample 1 was from patient 1 at 12 months after autologous CAR-T treatment, and sample 2 from patient 5 at 9 months after allogeneic CAR-T therapy. After performing quality control, we included 8627 cells from sample 1 and 12,455 cells from sample 2 for further analysis. UMAP analysis revealed 22 clusters in both sample 1 and 2 (Supplementary Figs. 5 and 6). Sample 2 contained a higher proportion of T cells compared with sample 1 (79.40% vs 55.04%). Among T cells, CD8^+^ T cells accounted for 74.27% in sample 2. A predominant cluster of NK cell accounted for 10.46% in sample 1 (Fig. 5A). Notably, CD7 expression differed significantly between the two samples, especially higher expression on NK cells from sample 1, but almost absent on sample 2 (Fig. 5B). We analyzed differentially expressed genes in CD4^+^ T, CD8^+^ T, and NK cells, and found that only CD4^+^ T in sample 1 highly expressed FOS, which was enriched in the TNF signaling pathway (Supplementary Figs. 7 and 8). CD7 and FCER1G were upregulated in NK cells in sample 1, but were not enriched to any pathway.

We further divided T cells into 16 clusters via Principal Component Analysis and found that 93.9% of T cells from sample 2 expressed T-cell effector function-related genes (Supplementary Figs. 9 and 10). Patient 1 had higher levels of memory T, γδ T and cytotoxic T cells with high granzyme B compared with patient 5 (Fig. 5C, D). Immune checkpoint, TIGIT, was widely expressed on effector T cells of patient 1 but limited to exhausted T cells of patient 5 (Fig. 5D). Patient 5 had relatively less regulatory T cells compared with patient 1, and myeloid-derived suppressor cells and tumor-associated macrophages were not detected.

## Discussion

Patients with relapsed or refractory T-ALL/lymphoma have dismal outcomes under the intervention of traditional chemotherapies and the combination of targeted medicines showed limited efficacy [34, 35]. Thus, CAR-T therapy were considered the last resort to save these patients. The products adopted in our trial has been reported in a previous study among children and youngers with T-ALL [22]. However, advanced age is a high-risk factor for poor prognosis for leukemia. In this clinical trial, we focus on the teenagers and adults with T-ALL or lymphoma. Among patients aging 16 to 69, 70% achieved CR with mild CRS and no ICANS.

Another hit of our study is to identify the pros and cons of CAR-T cells derived from different cell origins which can provide evidence to optimize the treatment. Although it seems that allogeneic CAR-T cells would not shorten the length of manufacture, allogeneic CAR-T cells do have advantages in acquiring the optimal window of the latest therapy. After the latest chemotherapy, patients with autologous CAR-T cells were demanded for more time to recover from cytopenia (median time, 36 days vs. 11 days). For patients with highly aggressive and rapidly progressing malignancies, the choice to receive allogeneic CAR-T cells can eliminate the need to go through a one-month drug elution period before leukapheresis, which can greatly reduce the likelihood of rapid disease progression.

The CR rate of patients receiving allogeneic cells was 80% and that of patients with autologous products was 40%. During the follow-up period, the relapse rate showed more remarkable differences. One patient (25%) with allogeneic CAR-T cells suffered CD7^−^ recurrence. One hundred percent of patients treated with autologous CAR-T cells relapsed whether in BM or in EMD. Patients experienced CD7^+^ recurrence, but CAR copies were not detectable in vivo. The less persistence of autologous CAR-T cells might contribute to the treatment failure. CAR-T cells could stably survive in 75% of patients with allogeneic cells but only 33% of patients receiving autologous cells at month 2. Single-cell RNA sequencing can further reveal the internal factors for the long-term persistence of allogeneic CAR-T cells. CAR copies did exist at month 9 of patient 5. The remaining anti-CD7 CAR-T cells spared CD7^-^ T and NK cells at the transcriptional level. Previous studies had demonstrated that a deficiency of CD7 might promote CD8^+^ T cells toward effector phenotype [36] and reduce secretion of interleukin-2 in CD4^+^ T cells [37], which could explain the downregulated expression of proliferation-related genes like FOS, as well as the decreased proportion of Treg cells. CD7 loss alleviates the exhaustion of CAR-T cells mediated by chronic antigen stimulation and reduces proportion of Treg cells in the immune microenvironment as the possible mechanism of long-term survival of allogeneic CAR-T cells

CRS, ICANS and GVHD are main adverse events but mild in our study. Patients with allogeneic CAR-T cells did not suffer from ICANS, severe CRS or GVHD. Expect for these common complications, hematological toxicities, HLH, infections and T-cell aplasia were also observed in this trial. Although hematological toxicities were significant, our data was broadly consistent with other anti-CD7 CAR-T studies [19–22]. CD34^+^ stem cell infusion and transplantation could effectively restore patients’ bone marrow hematopoietic function after multiple lines of treatment. Long-term cytopenia may also be associated with the absence of a bridging SCT. HLH has been described as a second inflammatory storm after CRS. The incidence of HLH in patients with CRS was 40.4% and 26.7% in anti-CD19 and CD22 CAR-T cells [38, 39]. Steroids, ruxolitinib and anakinra were recommended for the treatment of HLH. In our study, two patients developed HLH after CRS and were treated with steroids and etoposide. Heavy tumor burden has been proven high risks of HLH which may explain the deterioration in patient 10 with diffused gastrointestinal infiltration [38]. Two patients with EBV activation died of pneumonia on day 63 and day 75, respectively. EBV-associated B-cell lymphoproliferation has been reported in a case treated with anti-CD7 CAR-T cells [20]. Both of these clinical findings indicated that patients with a history of EBV infection should be enrolled in caution, otherwise they should be closely monitored. The recovery of T cells within one month was in line to the previous study. Single-cell RNA sequencing indicated that CD7^−^ T cells had normal immune functions [22].

Limitations exist in our study. This is not a randomized controlled trial in consideration of the patients’ conditions. Moreover, the sample size of patients is small and extended follow-up time are needed for further evaluation of long-term outcomes.

In summary, our study has revealed that this IntraBlock anti-CD7 CAR-T cell therapy had achieved remarkable efficacy in teenager and adult patients with R/R T-cell malignancies, especially for patients with allogeneic products. In face of high relapse rate of patients with autologous CAR-T cells, consolidation therapies are recommended and need further investigation. As the majority of adverse events common in these trials were manageable, the anti-CD7 CAR-T cell therapy has been regarded a promising option for patients with R/R T-cell malignancies.

## Supplementary information

Supplemental material

## Data Availability

The original contributions presented in the study are included in the article. The single-cell RNA sequencing analyzed in this study have been uploaded to GEO with an accession number, GSE227828. Further inquiries can be directed to the corresponding authors upon reasonable request.
