# Supplementary material for: Allogenic and autologous anti-CD7 CAR-T cell therapies in relapsed or refractory T-cell malignancies
Source: Blood Cancer J. 2023 Apr 25;13(1):61. doi: 10.1038/s41408-023-00822-w (PMC10125858; doi:10.1038/s41408-023-00822-w)
Supplement: Supplementary file 1 — Supplemental material [file 41408_2023_822_MOESM1_ESM.pdf]

SUPPLEMENTARY MATERIALS

Supplementary Methods

The inclusion and exclusion criteria of clinical trial

Inclusion Criteria:

1. Aged from 14 to 70 years;
2. Expected survival over 60 days;
3. Eastern Cooperative Oncology Group score 0-2;
4. Diagnosed as T-cell hematologic malignancies (including leukemia and lymphoma) according to WHO2016 criteria;
5. Patients must relapse or be refractory after at least two lines of therapy.
6. CD7 were positive in bone marrow or cerebrospinal fluid by immunohistochemistry or flow cytometry at screening, and one of the following conditions is satisfied:

   A. No remission was achieved after at least 2 lines of standard therapy;

   B. Relapse or progression after standard treatment;

   C. Relapse after autologous or allogeneic hematopoietic stem cell transplantation;
7. Have no fertility requirements or plans for one year since enrollment in this clinical trial;
8. Patient or his or her legal guardian voluntarily participates in and signs an informed consent form.

Exclusion Criteria:

1. Complicated with central system leukemia/lymphoma with active intracranial lesions;
2. Existing or preexisting CNS conditions, such as epileptic seizures, cerebrovascular ischemia/hemorrhage, dementia, cerebellar disease, or any CNS related autoimmune disease;
3. Symptomatic heart failure or severe arrhythmias;
4. Symptoms of severe respiratory failure;
5. Complicated with other types of malignant tumors;
6. Serum creatinine and/or urea nitrogen $\geq$ 1.5 times of normal value;
7. Suffer from sepsis or other uncontrollable infections;
8. Intracranial hypertension or brain consciousness disorder;
9. Severe mental disorders;
10. Have received organ transplantation (excluding bone marrow transplantation);
11. Female patients (fertile patients) had positive blood HCG test;
12. Hepatitis (including hepatitis B and C), AIDS and syphilis were screened positive;
13. Patients with graft-versus-host disease (GVHD) or who require immunosuppressant treatment;
14. The absolute value of lymphocytes was too low to manufacture CART cells;
15. Other conditions considered inappropriate by the researcher.

Single-cell RNA sequencing and analyses

Cell preparation

Having harvested peripheral blood mononuclear cells, cell count and viability was estimated using fluorescence Cell Analyzer (Countstar® Rigel S2) with AO/PI reagent after removal erythrocytes (Miltenyi 130-094-183) and then debris and dead cells removal was decided to be performed or not (Miltenyi 130-109-398/130-090-101). Finally fresh cells were washed twice in the RPMI1640 and then resuspended at 1×106 cells per ml in 1×PBS and 0.04% bovine serum albuminat.

Single cell RNA-seq library construction and sequencing

Single-cell RNA-seq libraries were prepared using SeekOne® DD Single Cell 5' library preparation kit (SeekGene Catalog No.). Briefly, appropriate number of cells were mixed with reverse transcription reagent and then loaded to the sample well in SeekOne® DD Chip S5. Subsequently Gel Beads and Partitioning Oil were dispensed into corresponding wells separately in chip. After emulsion droplet generation reverse transcription were performed at 53°C for 45 minutes and inactivated at 85°C for 5 minutes. Next, cDNA was purified from broken droplet and amplified in PCR reaction. The amplified cDNA product was fragmented, end repaired, A-tailed and ligated to sequencing adaptor. Finally the indexed PCR were performed to amplified the DNA representing 3' polyA part of expressing genes which also contained Cell Bar code and Unique Molecular Index. The indexed sequencing libraries were cleanup with SPRI beads, quantified by quantitative PCR (KAPA Biosystems KK4824) and then sequenced on illumina NovaSeq 6000 with PE150 read length.

Supplementary Figures

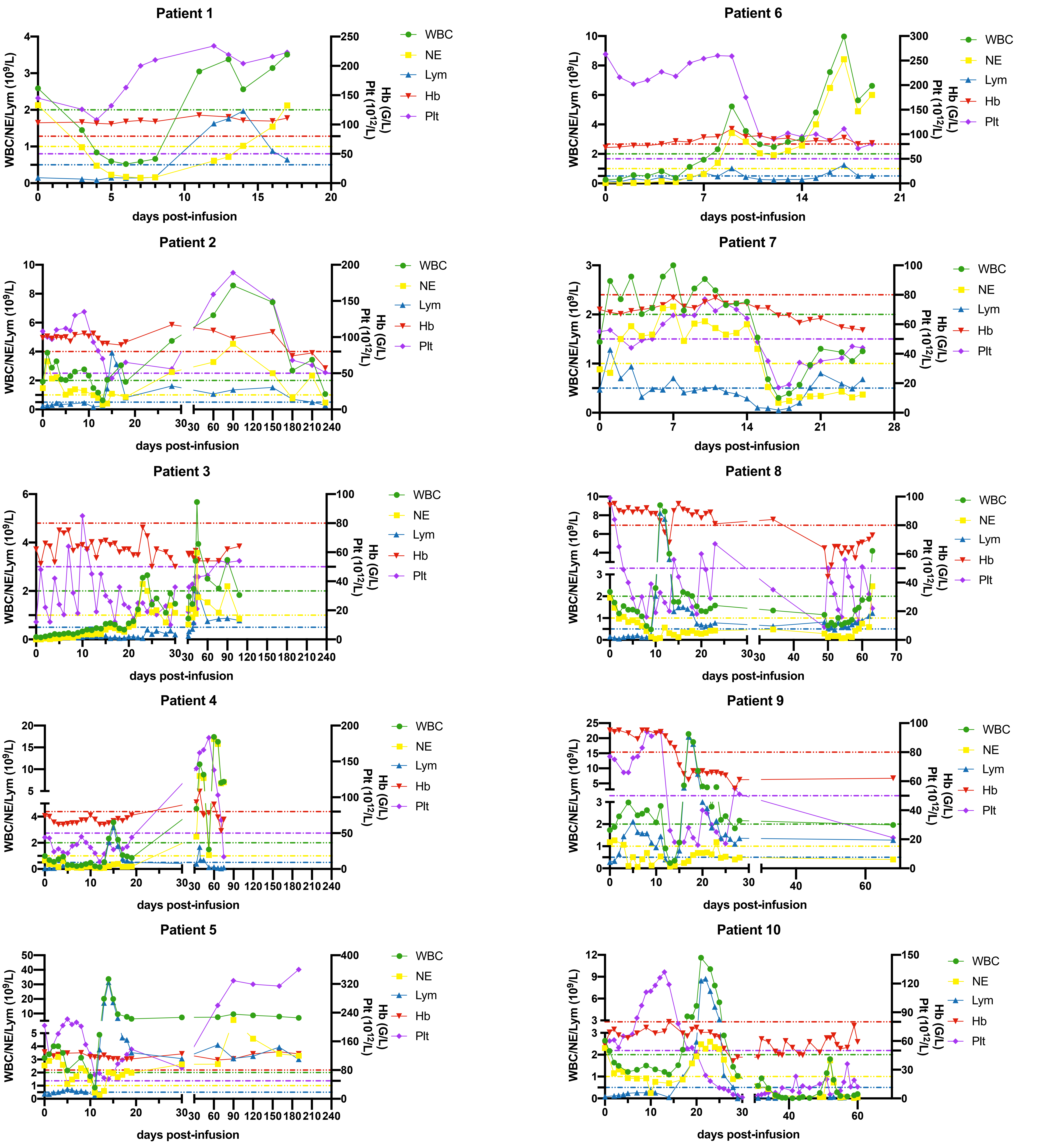

Supplementary Fig 1. Cytopenia within 30 days after infusions and prolonged hematological toxicities were presented in a plot per patient. Grids divided hematological toxicities into grade 1-2 and grade 3-4.

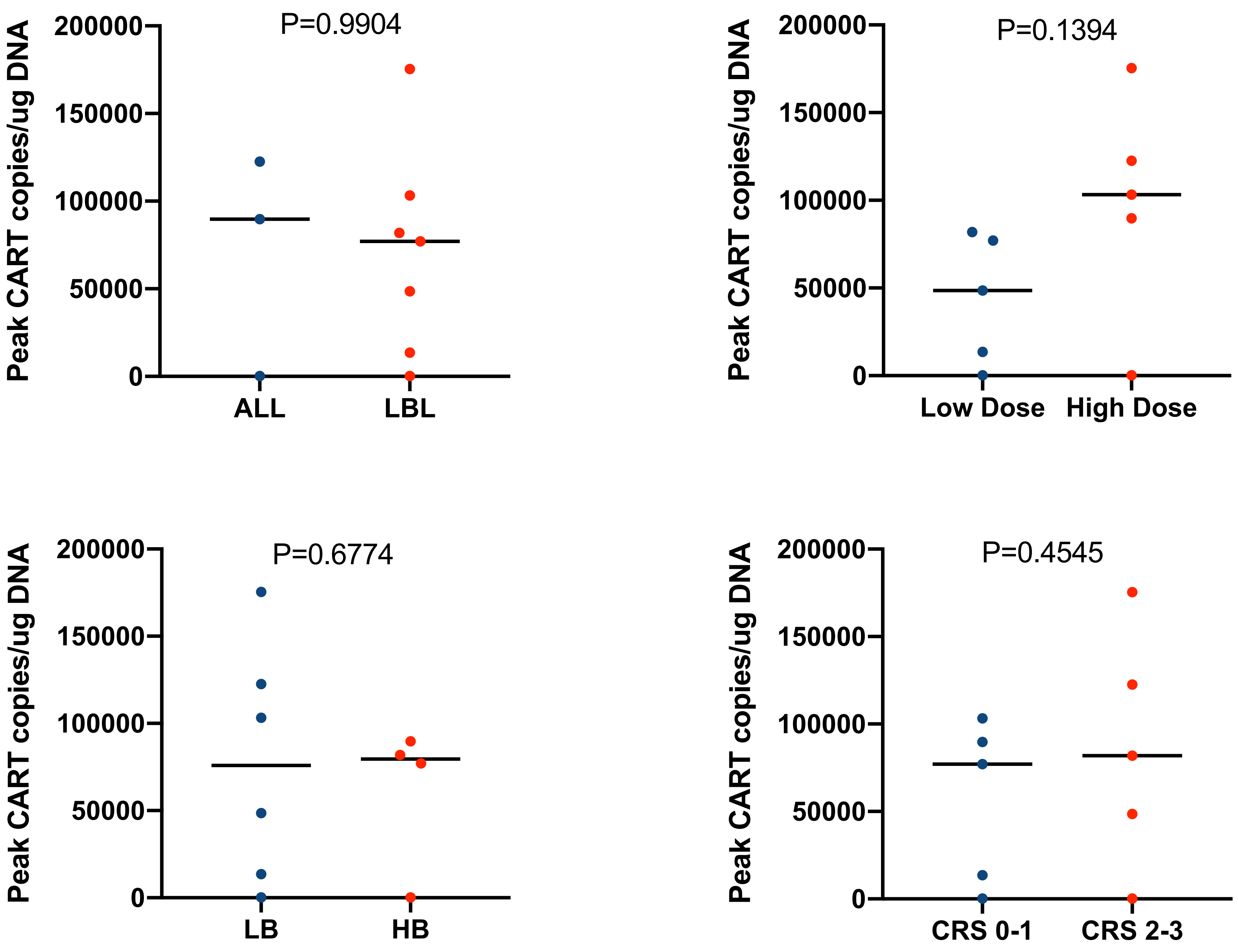

Supplementary Fig 2. Correlation of the peak CAR copies in the peripheral blood with sources, disease, tumor burden, doses, efficacy or adverse events. CRS, cytokine release symptom; LB: light tumor burden; HB: heavy tumor burden.

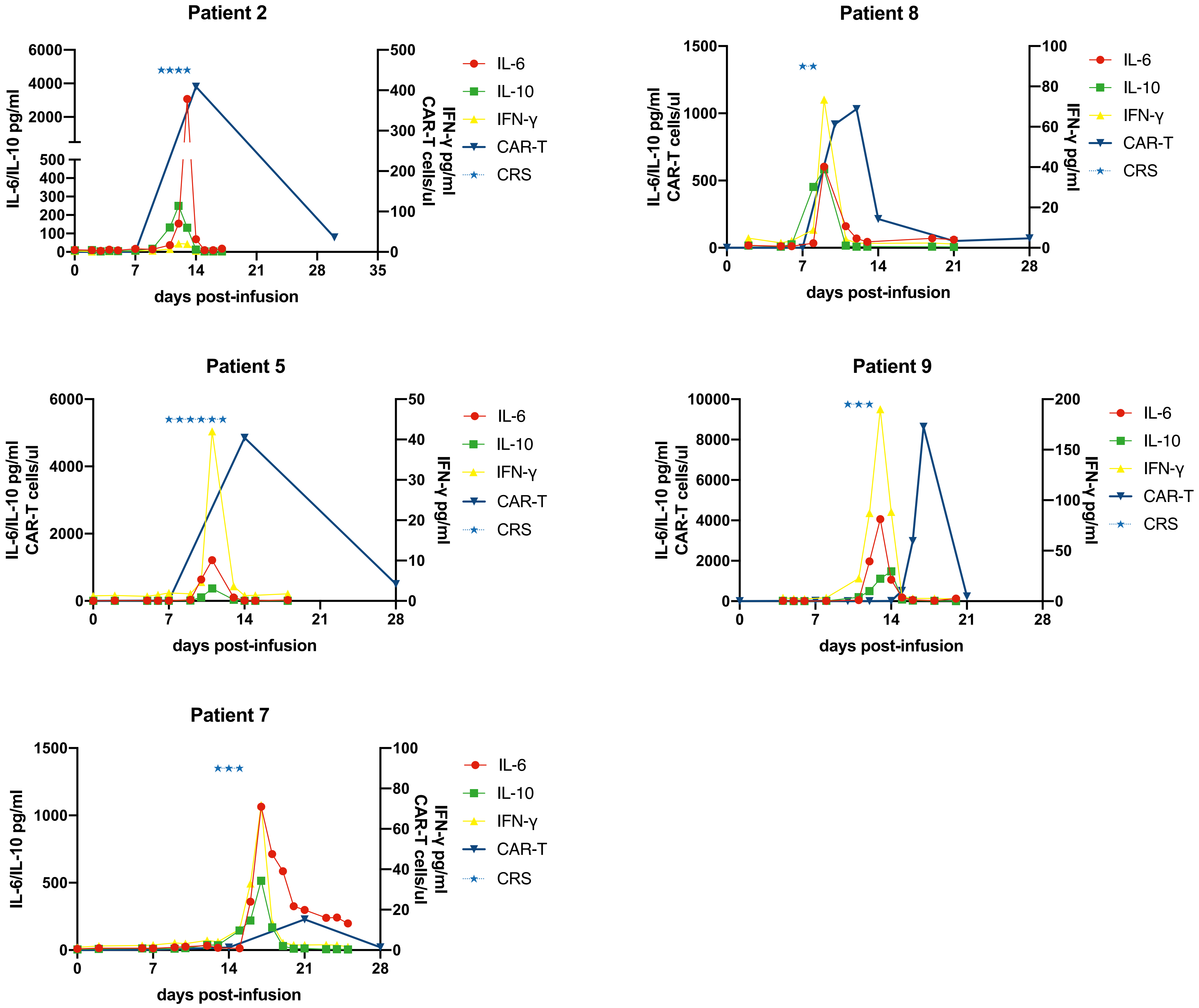

Supplementary Fig 3. In Patients 2, 5, 7, 8 and 9, CAR-T cell numbers (detected by flow cytometry), serum cytokine concentrations at indicated times post infusion and durations of cytokine release syndrome were shown.

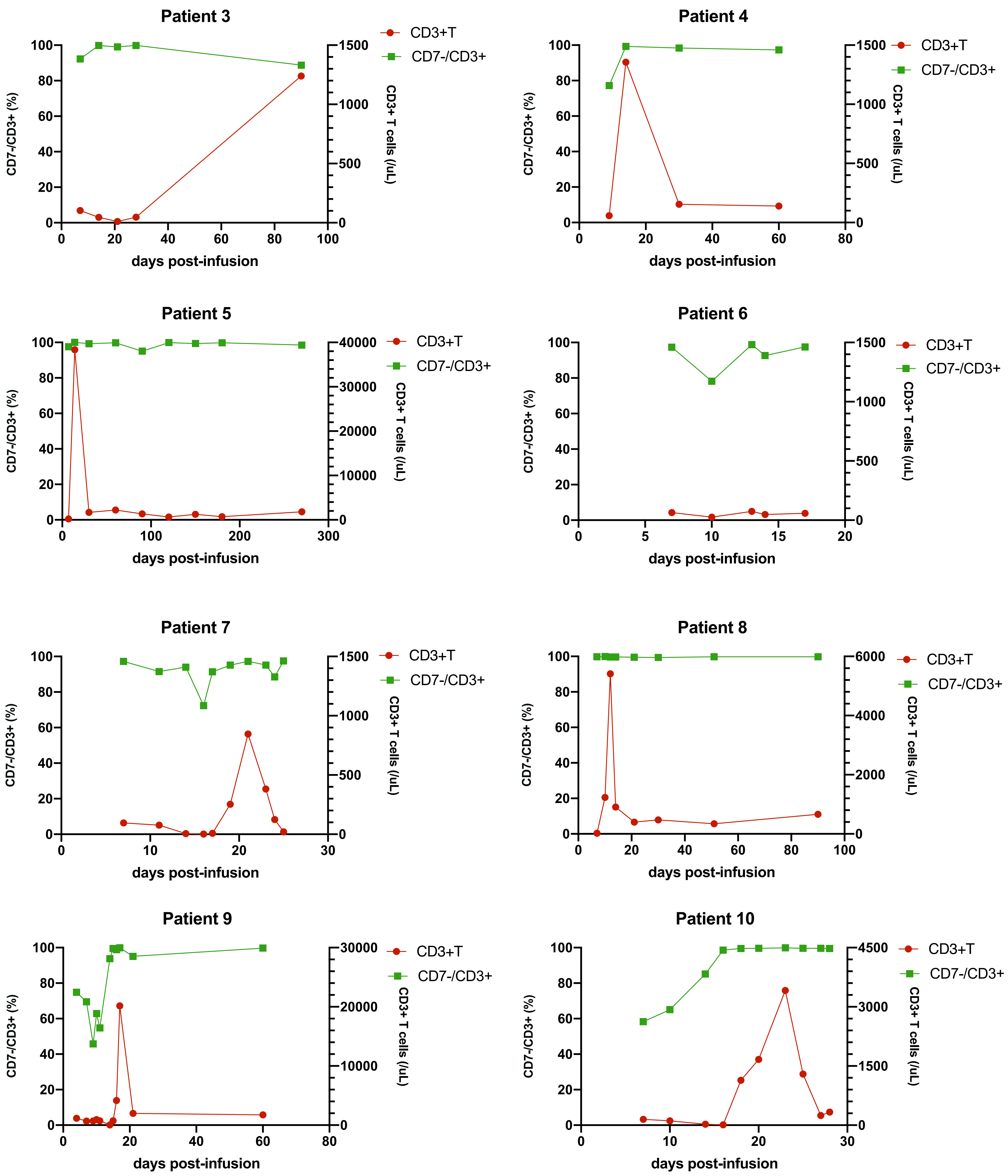

Supplementary Fig 4. Counts of $CD3^+$ T cell and proportions of $CD7^-$ T cells in $CD3^+$ T cells post-infusion.

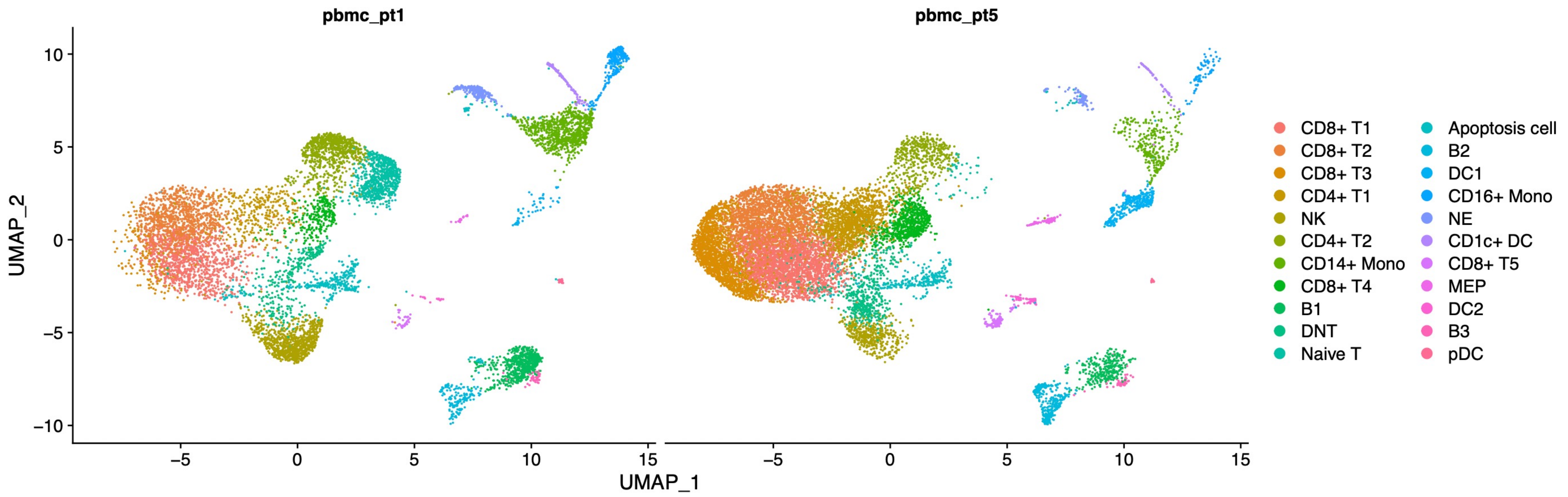

Supplementary Fig 5. Visualization of peripheral blood cells of patient 1 and patient 5 via UMAP.

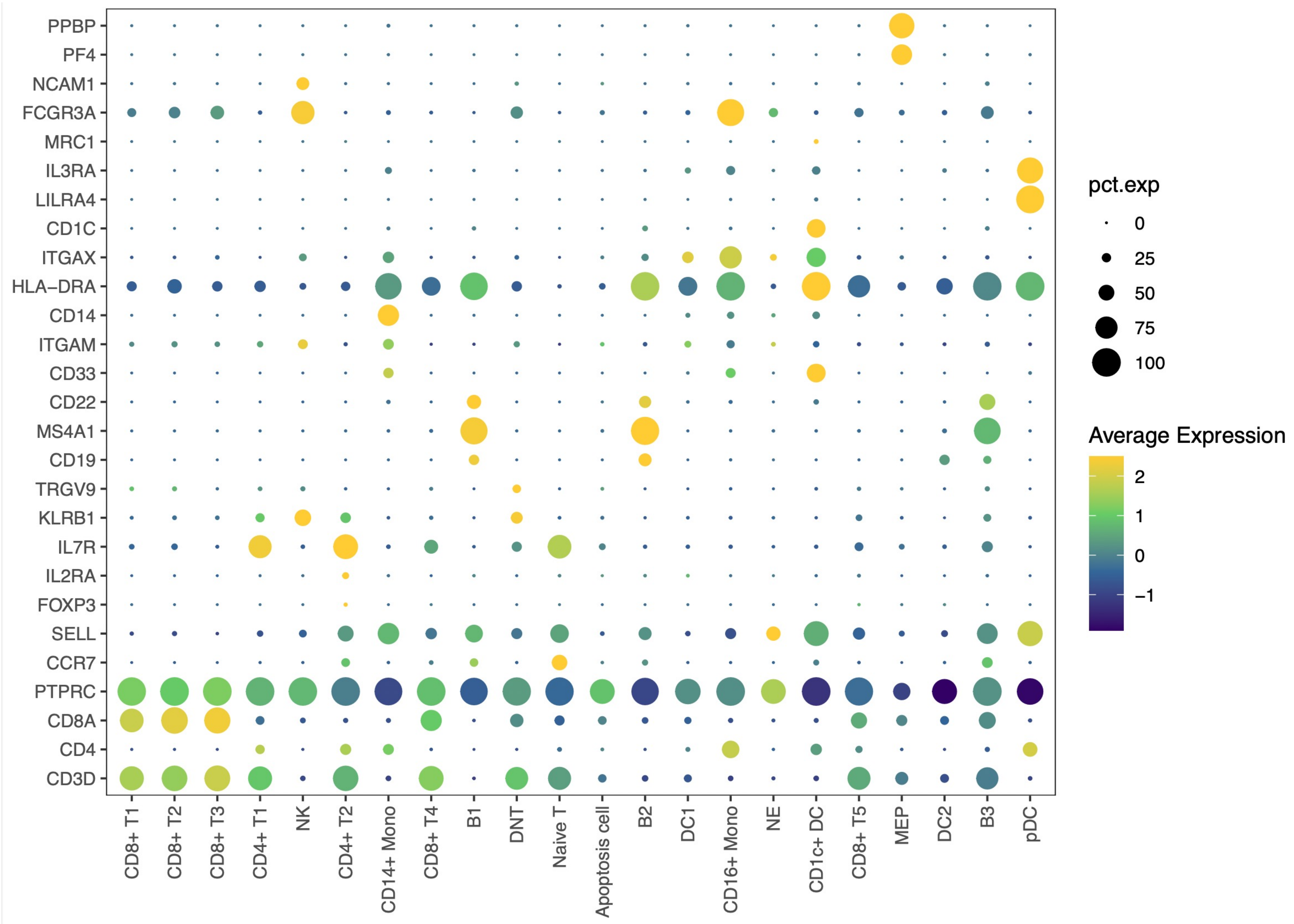

Supplementary Fig 6. The expression of marker genes for annotating cell subsets.

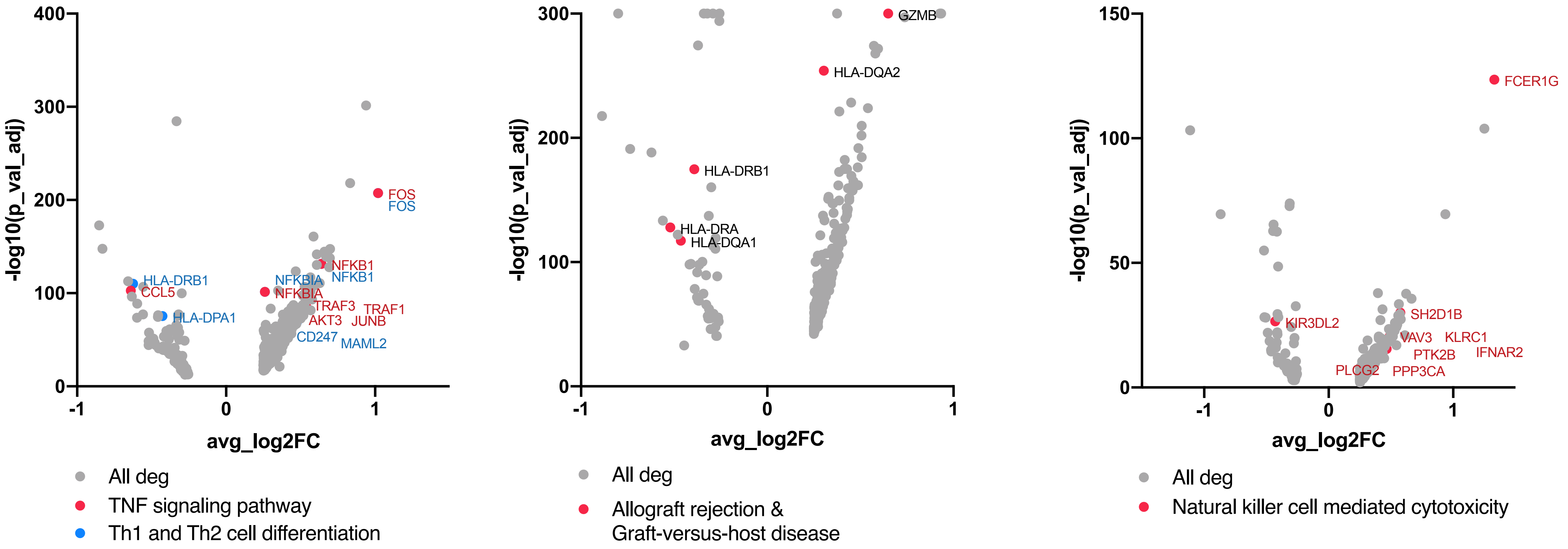

Supplementary Fig 7. Differential expressed genes of CD4+ T (left), CD8+T (middle), NK (right) between two samples. Associated genes of pathways enriched by KEGG database are indicated by colored dots.

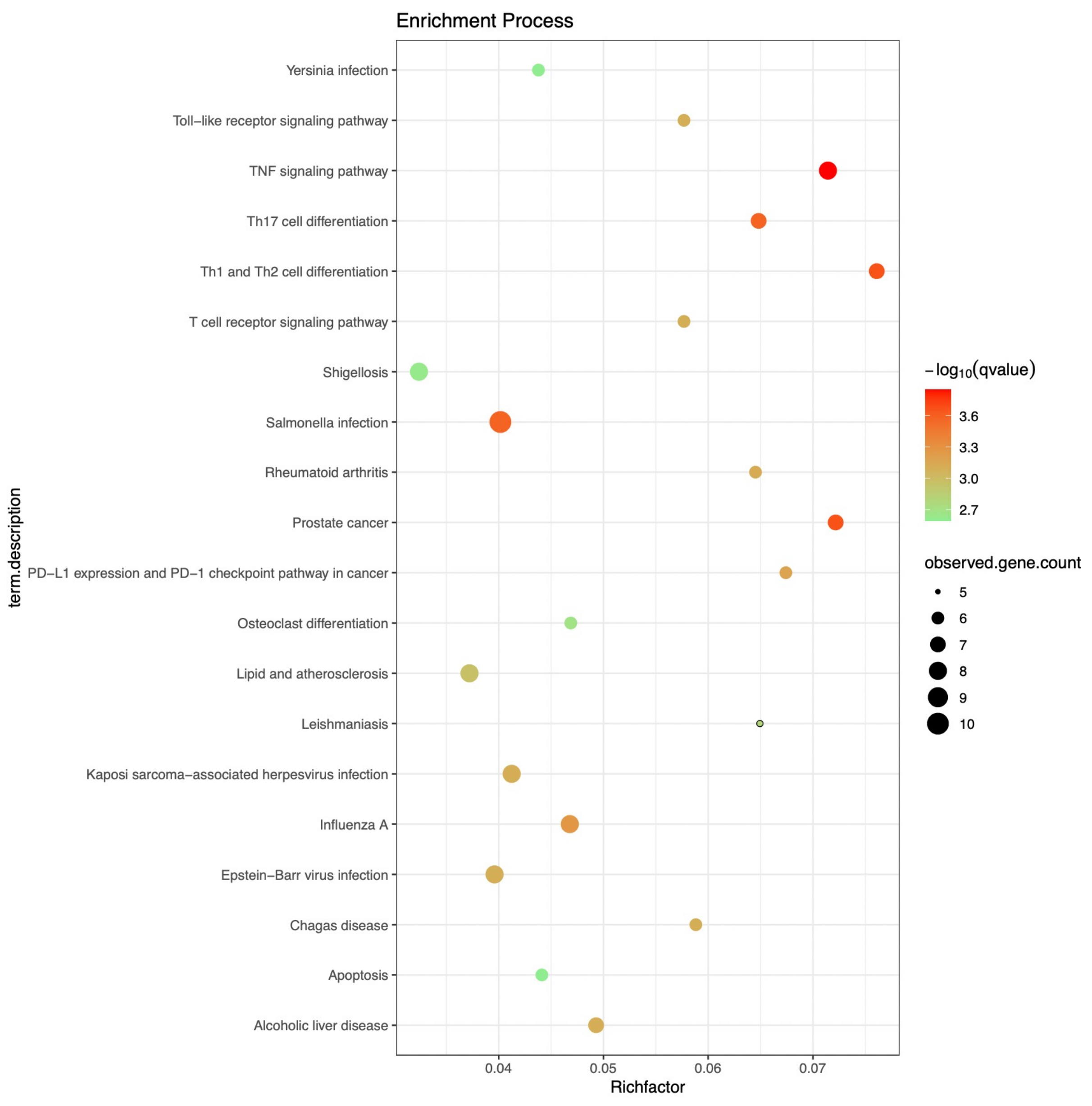

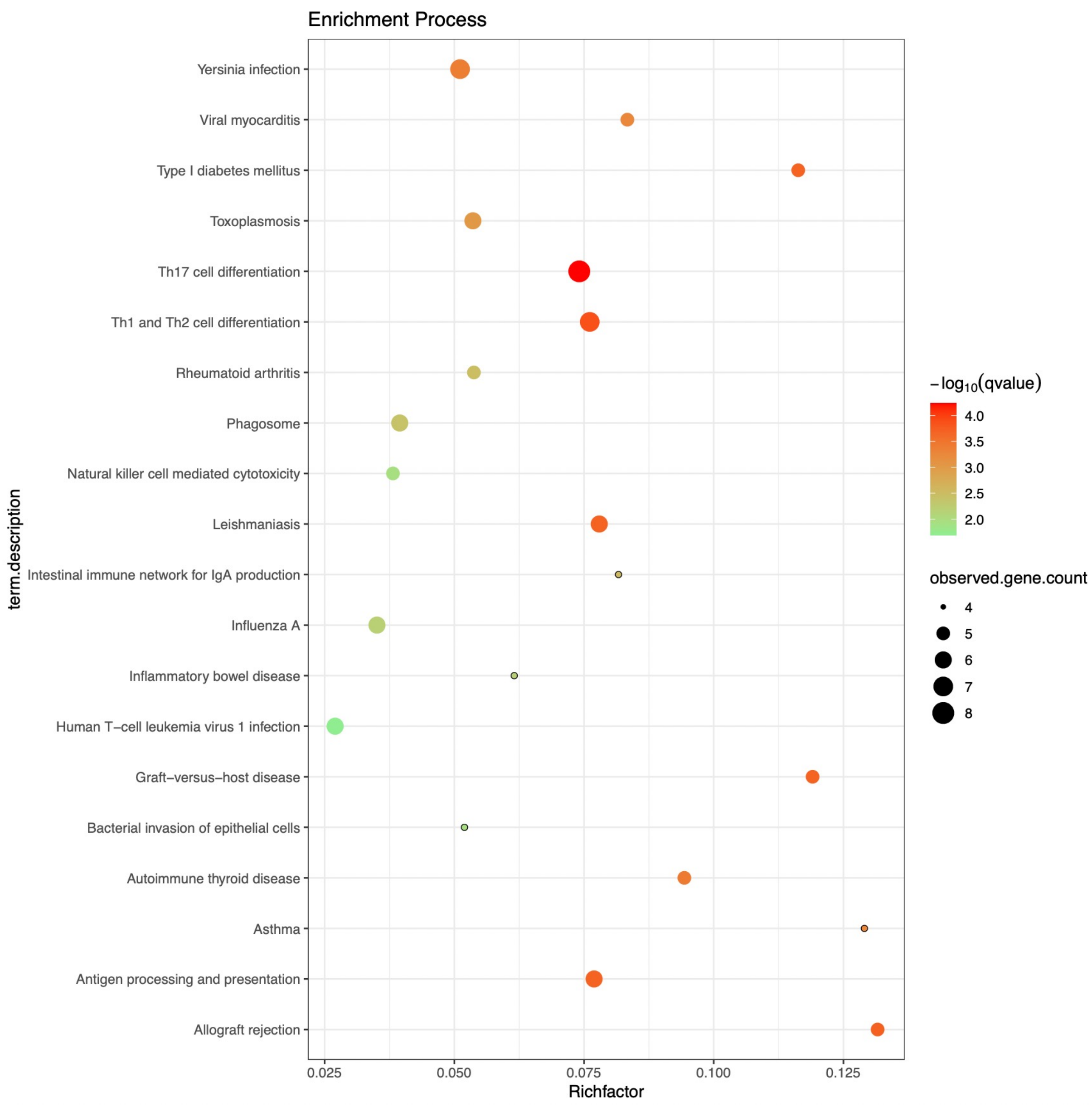

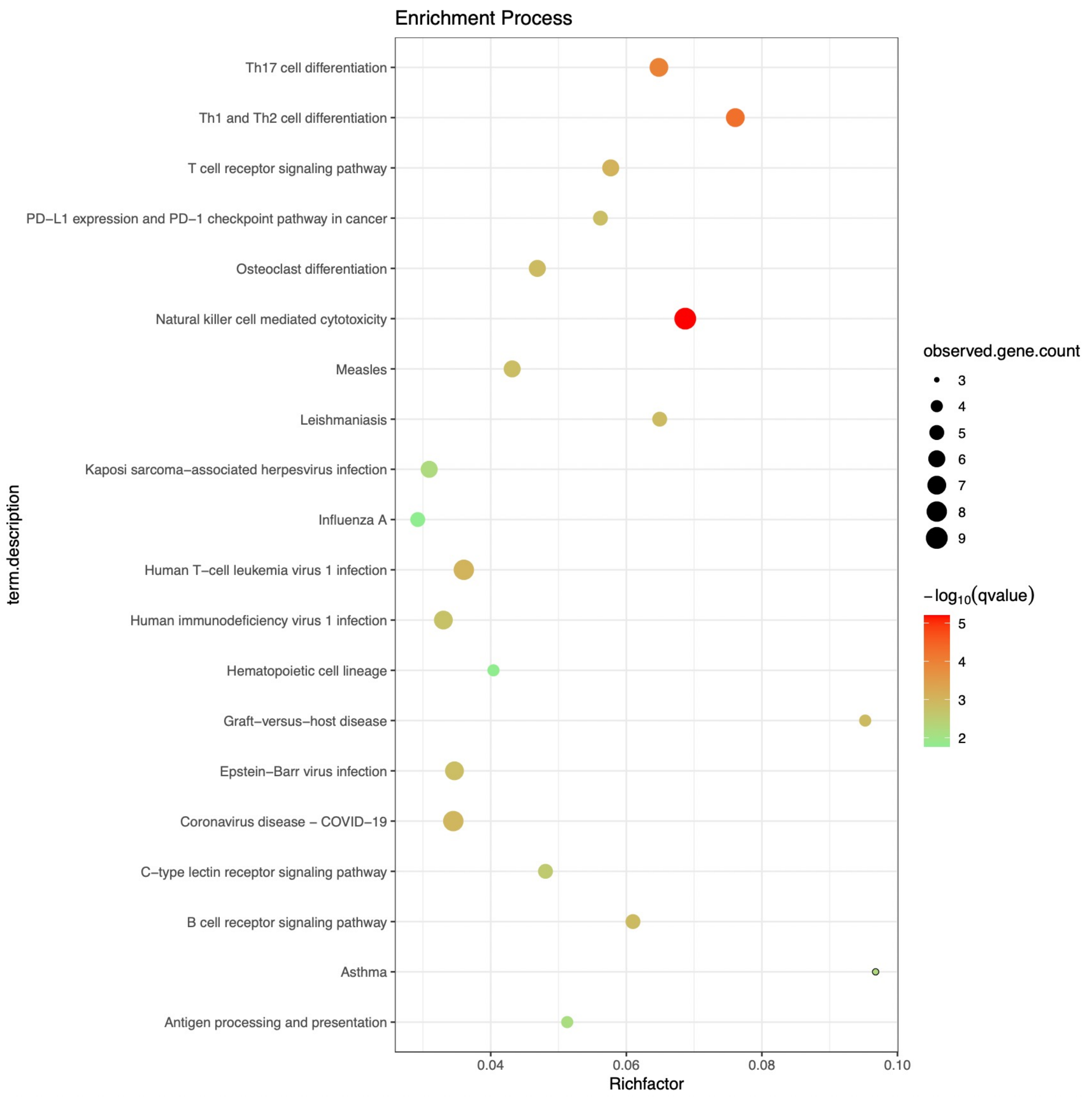

Supplementary Fig 8. Differential expression genes were enriched based on KEGG database. Upper left, CD4⁺ T; upper right, CD8⁺ T; lower left, NK.

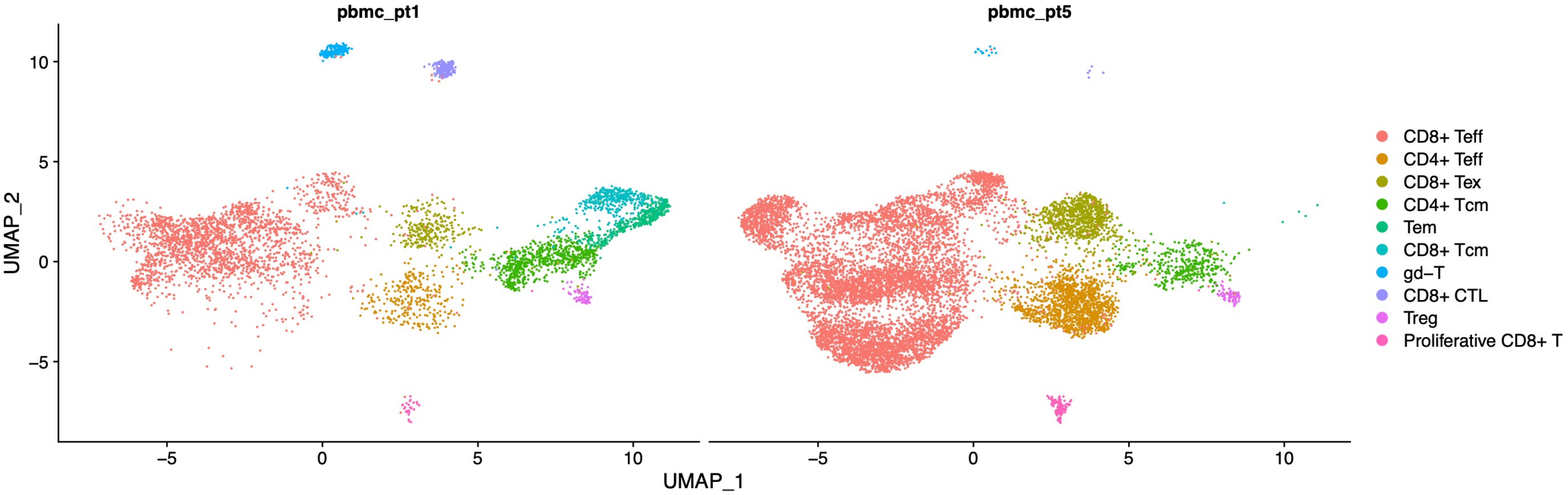

Supplementary Fig 9. Visualization of T cells of patient 1 and patient 5 via UMAP.

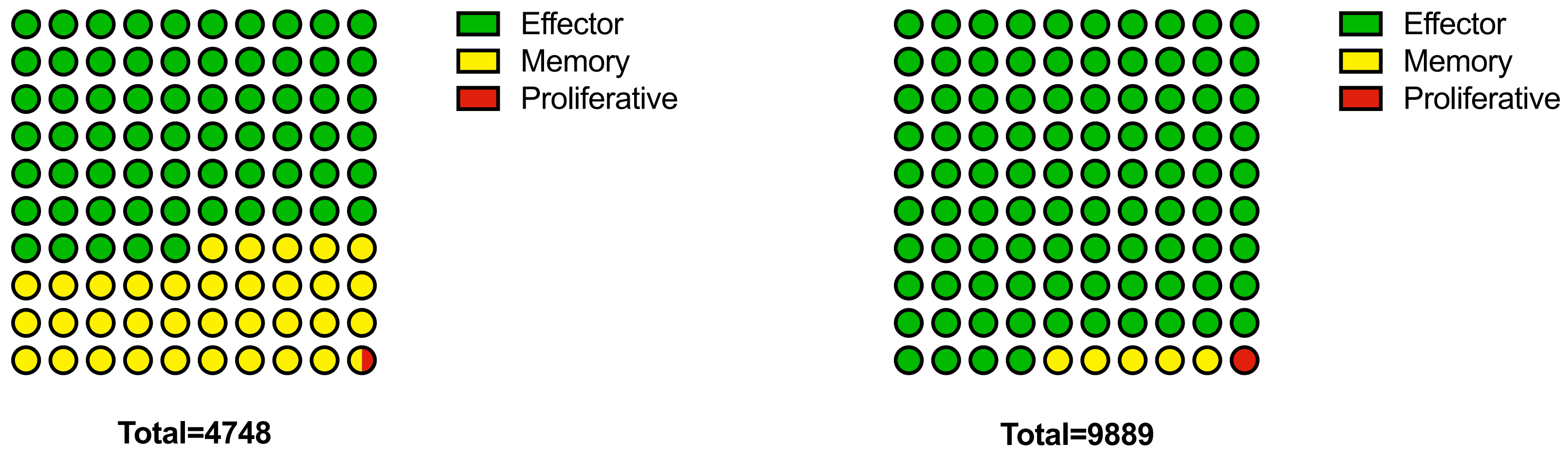

Supplement Fig 10. Distribution of effector, memory and proliferative T cells in patient 1 (left) and patient 5 (right).

Supplementary Tables

Supplemental Table 1. Characteristics of infusion products

	PBMCs source	viability of PBMCs (%)	raletive MFI in PBMCs	raletive MFI in products	CD4:CD8 ratio in PBMCs	CD4:CD8 ratio of CAR-T cells	folds of expansion	CAR+ cells (%)	infused CAR-T cells (1*106/kg)	products
PT1	patient	94.8	127.37	1.23	1.46	7.81	16.3	65.1	1.5	frozen
PT2	patient	97.3	224.40	1.65	0.91	7.11	10.3	81.7	2.5	frozen
PT3	patient	97.7	22.54	1.44	0.60	53.72	3.4	40.2	1.5	frozen
PT4	donor	100	304.47	1.57	1.20	8.01	14.9	71.2	2.5	frozen
PT5	donor	99.7	439.26	1.40	1.59	1.98	81.0	51.6	1.5	frozen
PT6	patient	100	424.52	1.47	0.29	1.51	14.0	46.5	2.0	frozen
PT7	donor	94.8	187.19	1.28	1.90	5.89	15.4	45.0	2.0	frozen
PT8	donor	96.4	475.69	1.35	2.01	3.38	12.4	46.0	1.0	frozen
PT9	donor	98.7	345.46	1.24	2.92	55.92	9.0	43.3	1.5	frozen
PT10	patient	97.7	275.77	1.26	0.75	5.77	13.3	40.7	1.0	frozen

MFI, mean fluorescence intensity

Relative MFI = (MFI of PE-CD7) / (MFI of PE-IgG1) in T cells

Supplemental Table 2. The windows for CAR-T manufaturing

		The latest therapy	From latest therapy/relapse to leukapheresis	Time of manufacture	From leukapheresis to infusion	Causes of delayed infusion
	PT1	chemotherapy	39	9	26	delayed admission due to the sarurated ward
	PT2	chemotherapy	33	8	19	delayed admission due to COVID-19
Autologous	PT3	HSCT	16	11	14	/
	PT6	chemotherapy	46	10	18	chidamide as bridging therapy for progressive disease
	PT10	chemotherapy	32	10	16	spot-check of the CAR-T products
	PT4	stem cells infusion	52	10	22	controlling HSCT-related GVHD
	PT5	HSCT	23	10	26	controlling conjuntivitis
Allogeniec	PT7	chemotherapy	16	9	17	chidamide as bridging therapy for progressive disease
	PT8	HSCT	25	9	27	delayed admission due to COVID-19
	PT9	chemotherapy	6	10	101*	/

* the patient achieved CR when the product was done and received CAR-T cells after his second relapse.

Supplemental Table 3. Detailed information of adverse events

	CRS					Infection				GVHD		Others
	Garde	Symptoms	Onset	Duration	Treatment	Types	Pathogene	Location	Onset	Grade	symptoms	
Patient1	0	/	/	/	/							
Patient2	2	fever, hypoxia, hypotension	D10	4	Tocilizumab 320mg. Dexamethasone 42.5mg	Virus Fungus	EBV C tropicalis	Pneumonia	D102 D181	1	cGVHD: hyperpigmentation	Capillary leak syndrome, hypofibrinogenemia
Patient3	3	fever, hypoxia, hypotension	D15	9	Dexamethasone 15mg	Bacteria	ABA	Unknown	D19			HLH
Patient4	1	fever	D10	3	Dexamethasone 30mg	Bacteria Virus	SE EBV, CMV	Pneumonia	D3 D62	2	aGVHD: rash, diarrhea	
Patient5	1	fever, myalgia	D7	6	Tocilizumab 960mg. Dexamethasone 67.5mg							Hypofibrinogenemia
Patient6	0					Bacteria	SMA	Pneumonia	D9			
Patient7	1	fever	D13	3	Tocilizumab 400mg. Dexamethasone 22.5mg							Capillary leak syndrome, hypofibrinogenemia
Patient8	2	fever, hypoxia	D7	2	Tocilizumab 320mg. Dexamethasone 22.5mg	Virus	CMV	Unknown	D43			Hypofibrinogenemia
Patient9	2	fever, hypoxia	D10	4	Tocilizumab 960mg. Dexamethasone 92.5mg							Hypofibrinogenemia
Patient10	2	fever, hypotension	D13	5	Dexamethasone 95mg	Virus Fungus	HPV6, EBV, CMV TA	Pneumonia	D27			HLH

CRS, cytokine release symdrome; EBV, Epstein-Barr virus; ABA, Acinetobacter baumannii; SE, Staphylococcus epidermidis; CMV, cytomegalovirus; SMA, Stenotrophomonas maltophilia; HPV, Human papillomavirus; TA, Trichosporon asahiia; GVHD, acute graft-versus-host disease; cGVHD, chronic graft-versus-host disease; HLH, hemophagocytic lymphohistiocytosis

Supplemental Table 4. Grades of cytopenia pre-FC, pre-infusion and post-infusion

	Anemia			Thrombocytopenia			Leukopenia			Neutropenia			Lymphocytopenia		
	pre-FC	pre-infusion	post-infusion	pre-FC	pre-infusion	post-infusion	pre-FC	pre-infusion	post-infusion	pre-FC	pre-infusion	post-infusion	pre-FC	pre-infusion	post-infusion
Patient1	1	1	1	0	0	1	2	2	4	0	0	4	2	4	4
Patient2	1	1	1	1	1	3	0	3	4	0	2	4	0	0	4
Patient3	4	4	4	4	4	4	4	4	4	4	4	4	3	4	4
Patient4	2	3	3	3	3	4	3	4	4	3	3	4	3	4	4
Patient5	0	1	1	0	0	2	0	1	4	0	0	4	0	3	4
Patient6	3	3	3	0	0	1	0	4	4	0	4	4	2	4	4
Patient7	3	3	3	3	3	4	0	3	4	0	3	4	0	4	4
Patient8	1	2	3	1	2	4	0	2	4	2	3	4	0	4	4
Patient9	2	2	3	1	1	4	0	3	4	0	2	4	0	3	4
Patient10	3	3	3	1	2	4	3	2	4	3	3	4	2	4	4

FC, fludarabine and cyclophosphamide

Supplemental Table 5. Causes of death for each groups

		Follow-up outcomes	Causes of Death
Autologous	PT1	sustained CR on M14	/
	PT2	died on M7	Relapsed on D139 and died of progressive disease
	PT3	died on M6	Relaplsed on D103 and died of progressive disease
	PT6	withdraw on D19	/
	PT10	died on D63	Developed EBV-related HLH and died of fungal pneumonia
Allogeniec	PT4	died on D75	Died of EBV+ pneumonia
	PT5	sustained CR on M9	/
	PT6	withdraw on D25	/
	PT8	died on M5	Relapsed on D80 and died of progressive disease
	PT9	died on D81	Died from a sudden intracerebral hemorrhage
